# Green spectrofluorimetric quantification of aspirin, olmesartan, and metoprolol in spiked human plasma

**DOI:** 10.1038/s41598-023-46042-x

**Published:** 2023-11-17

**Authors:** Ali Alqahtani, Taha Alqahtani, Reem M. Gahtani, Sherif Ramzy

**Affiliations:** 1 Department of Pharmacology, College of Pharmacy, King Khalid University, 62529 Abha, Saudi Arabia; 2 Department of Clinical Laboratory Sciences, College of Applied Medical Sciences, King Khalid University, 61421 Abha, Saudi Arabia; 3 Pharmaceutical Analytical Chemistry Department, Faculty of Pharmacy, Al-Azhar University, Cairo, 11751 Egypt

**Keywords:** Analytical chemistry, Green chemistry

## Abstract

Low dose aspirin is routinely taken with antihypertensive drugs such as olmesartan and metoprolol to avoid the cardiovascular and renal outcomes associated with high blood pressure. The first spectrofluorimetric method for quantifying aspirin, olmesartan, and metoprolol in spiked human plasma is described here. The emission/excitation wavelengths of Aspirin, olmesartan, and metoprolol were 404 nm/290 nm, 372 nm/250 nm, and 302 nm/230 nm, respectively. The native fluorescence spectra of metoprolol do not overlap with those of aspirin or olmesartan, although the spectra of aspirin and olmesartan overlap. As a result, metoprolol could be measured directly in a mixture at 302 nm following excitation at 230 nm. Using synchronous fluorescence spectrometry at Δλ = 110 allowed for the determination of olmesartan at 364 nm with no interference from aspirin and metoprolol. Coupling the synchronous fluorescence spectrometry with second-order derivative allowed for the determination of aspirin at 426 nm with no interference from olmesartan and metoprolol. The suggested approach has been validated using ICH M10 criteria for bioanalytical method validation and was effectively utilized for quantification of tested medications in human plasma with reasonable accuracy and precision findings. Furthermore, using two greenness metrics, the Green Analytical Procedure Index and the Analytical GREEnness, the suggested method obtained a high greenness score.

## Introduction

**Figure 1:**
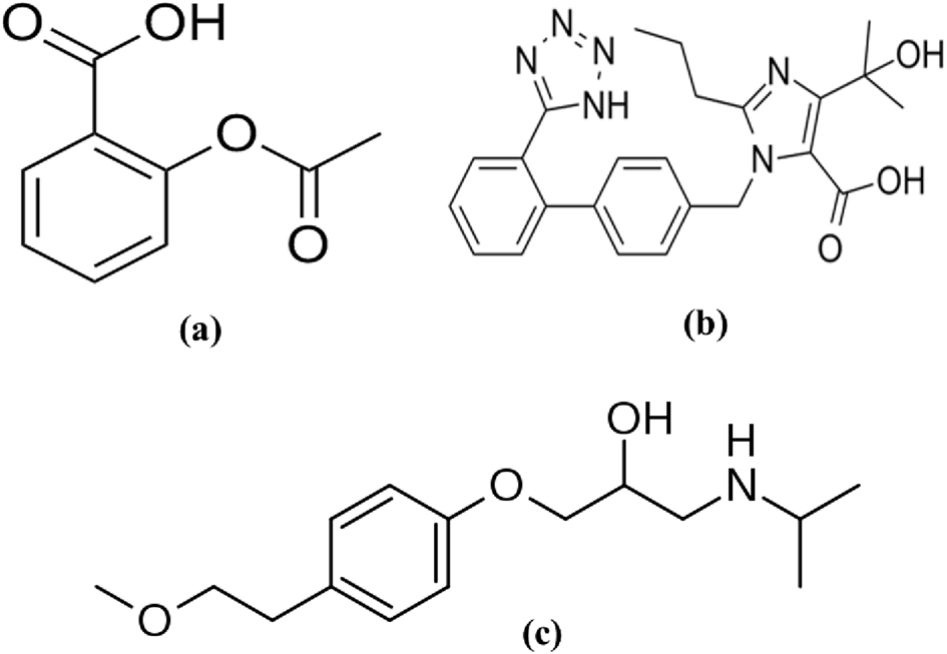
Chemical structures of ASA (**a**), OST (**b**), and MPL (**c**).

Low dose aspirin, also known as acetylsalicylic acid (ASA), is routinely taken with antihypertensive drugs to avoid the cardiovascular and renal outcomes associated with high blood pressure^1^. Olmesartan (chemically named 5-(2-hydroxypropan-2-yl)-2-propyl-3-[[4-[2-(2H-tetrazol-5-yl)phenyl]phenyl]methyl]imidazole-4-carboxylic acid) and metoprolol (chemically named 1-[4-(2-methoxyethyl)phenoxy]-3-(propan-2-ylamino)propan-2-ol) are two antihypertensive medications with different mechanisms that were recently combined as a fixed dosage combination. Olmesartan (OST) blocks the angiotensin II receptor, whereas metoprolol (MPL) blocks the beta_1_-adrenergic receptor^2,3^. Various analytical approaches for analyzing OST and MPL in combination have been documented in the literature^4–11^. ASA, OST, and MPL (Fig. 1) are commonly administered together for hypertension patients, thus developing an analytical method sensitive enough to determine them in human plasma is significant.

The fundamental goal of this research is to provide a simple, sensitive, and green spectrofluorimetric method for detecting ASA, OST, and MPL in human plasma. It is worth mentioning that in plasma, ASA is rapidly metabolized to salicylic acid, which has the same spectral characteristics as ASA^12,13^. The emission/excitation wavelengths of ASA, OST, and MPL were 404 nm/290 nm, 372 nm/250 nm, and 302 nm/230 nm, respectively. The MPL fluorescence emission spectra at 302 nm were completely resolved from the ASA, and OST spectra. As a result, without interference from ASA or OST, MPL could be determined directly in mixture at 302 nm. ASA, and OST fluorescence emission spectra, on the other hand, were overlapping, making direct native determination difficult. Measuring the synchronous fluorescence spectra of the studied drugs using Δλ = 110 allowed for the determination of OST at 364 nm with no interference from ASA and MPL. Transformation of the synchronous fluorescence spectra into a second-order derivative allowed for the determination of ASA at 426 nm with no interference from OST, and MPL. Using ICH M10 criteria for bioanalytical method validation, the proposed approach has been proven to deliver high accuracy and precision in quantifying tested drugs in spiked human plasma. To assess the greenness of the applied method, Green Analytical Procedure Index (GAPI)^14^ and Analytical GREEnness (AGREE)^15,16^ have been applied. Based on the results, the applied method adheres very closely to the principles of Green Analytical Chemistry.

## Experimental

### Materials and reagents

ASA, OST, and MPL powders were supplied by the Egyptian Drug Authority. Human plasma from different sources was supplied by the National Egyptian Blood Bank. We used HPLC-grade solvents and double-distilled water during the procedure. Analytical-grade chemicals and reagents were employed in the analysis procedure.

### Instrumentation

All measurements were performed with a Jasco FP-6200 spectrofluorometer (Tokyo, Japan). The measured spectra were manipulated using Jasco Spectra Manager software.

### Preparation of the solutions

Standard solutions (100 µg/mL) of ASA, OST, and MPL were generated by mixing 10 mg of every medication powder in an independent 100-mL volumetric flask with 25 mL ethanol, shaking well, and filling to capacity with water. Further dilutions with water were made to create a series of working solutions for each medication, with concentrations ranging from 0.2 to 10 µg/mL for ASA, 0.5–8 µg/mL for OST, and 1–14 µg/mL for MPL.

### Method development and validation

#### Calibration curves and range

To three separate sets of centrifuge tubes, 0.1 mL of human plasma was put along with 5 mL of acetonitrile. Afterwards, the tubes were spiked with 1 mL of the working standard solution of the studied drugs and vortexed for 10 min before centrifugation at 5000 rpm for 20 min. Immediately following drying of the supernatants, the residues were mixed with ethanol and placed in three independent series of 10-mL volumetric flasks. The flasks were vigorously stirred after adding 1 mL of acetate buffer solution (pH 5) to each. The volume of each flask was then adjusted with water to provide seven calibration samples for each drug, with concentrations ranging from (defined by LLOQ “lower limit of quantification” and ULOQ “upper limit of quantification”) 20–1000 ng/mL for ASA, 50–800 ng/mL for OST, and 100–1400 ng/mL for MPL.

The samples were measured in spectrofluorometer ordinary mode, and MPL was detected directly at 302 nm following excitation at 230 nm, with no interference from ASA or OST. The MPL calibration curve was created by graphing the fluorescence intensity at 302 nm versus the matching MPL concentration. The solutions were then measured in spectrofluorometer using the synchronous settings at ∆λ = 110 nm to detect OST directly at 364 nm with no contribution from ASA or MPL. The synchronous intensities at 364 nm were graphed against the matching OST concentration to produce the OST calibration curve. The synchronous spectra of the tested medications were then transformed into a second-order derivative in which ASA might be detected directly at 426 nm with no contribution from OST or MPL. The ASA calibration curve was created by graphing the peak amplitudes at 426 nm versus the matching ASA concentration.

#### Selectivity

The capacity of the used approach to selectively quantify the measured medications in the presence of endogenous interferent of the matrix was examined. This was accomplished by analysing blank samples prepared from pooled plasma collected from six separate sources to investigate any fluorescence responses from interfering components at the fluorescence responses of the studied drugs.

#### Accuracy and precision

Four quality control (QC) samples of different concentration levels were prepared separately for each drug using the procedure mentioned under calibration curves and range. The four concentration levels were the LLOQ, within three times of the LLOQ (low QC), 30–50% of the calibration range (medium QC) and at least 75% of the ULOQ (high QC). Each sample was measured in five replicates on the same day for intra-day accuracy and precision, and on 3 days for inter-day accuracy and precision. The precision was determined as a percent coefficient of variation (%CV), but the accuracy was calculated as a mean percent recovery.

#### Matrix effect

The effect of unknown substances in the plasma matrix on the drugs fluorescence was evaluated by analyzing in triplicate low QC and high QC samples prepared from six different plasma sources. For each plasma matrix source, the accuracy and precision were determined.

#### Extraction recovery

Low, medium, and high QC samples were utilized during extraction recovery studies. The samples were prepared from six different plasma sources and processed in two sets. The first set is called the pre-spiked set, where the drugs standards were spiked into plasma before the extraction process was performed. The second set is called the post-spiked set, where the drug standards were spiked into the plasma after the extraction process was performed. Each sample was analyzed in triplicate. The extraction recovery was calculated using the following equation:

$$\% {\text{ Extraction}}\;{\text{Recovery}}\, = \,\left[ {\left( {{\text{Response}}\;{\text{of}}\;{\text{pre - Spike}}} \right)/\left( {{\text{Response}}\;{\text{of}}\;{\text{post - Spikes}}} \right)} \right] \times 100.$$

#### Stability

Fresh low QC and high QC samples were made in triplicate to investigate the stability of the studied drugs in the biological matrix. The samples were tested for bench top stability after 3 h at room temperature (RT) and freeze–thaw matrix stability after three cycles of freezing (at least 12 h) and thawing.

### Ethics approval and consent to participate

This work was approved by the Committee of Research Ethics in the Faculty of Pharmacy, Al-Azhar University, Cairo, Egypt.

## Results and discussion

### Spectral characteristics

**Figure 2:**
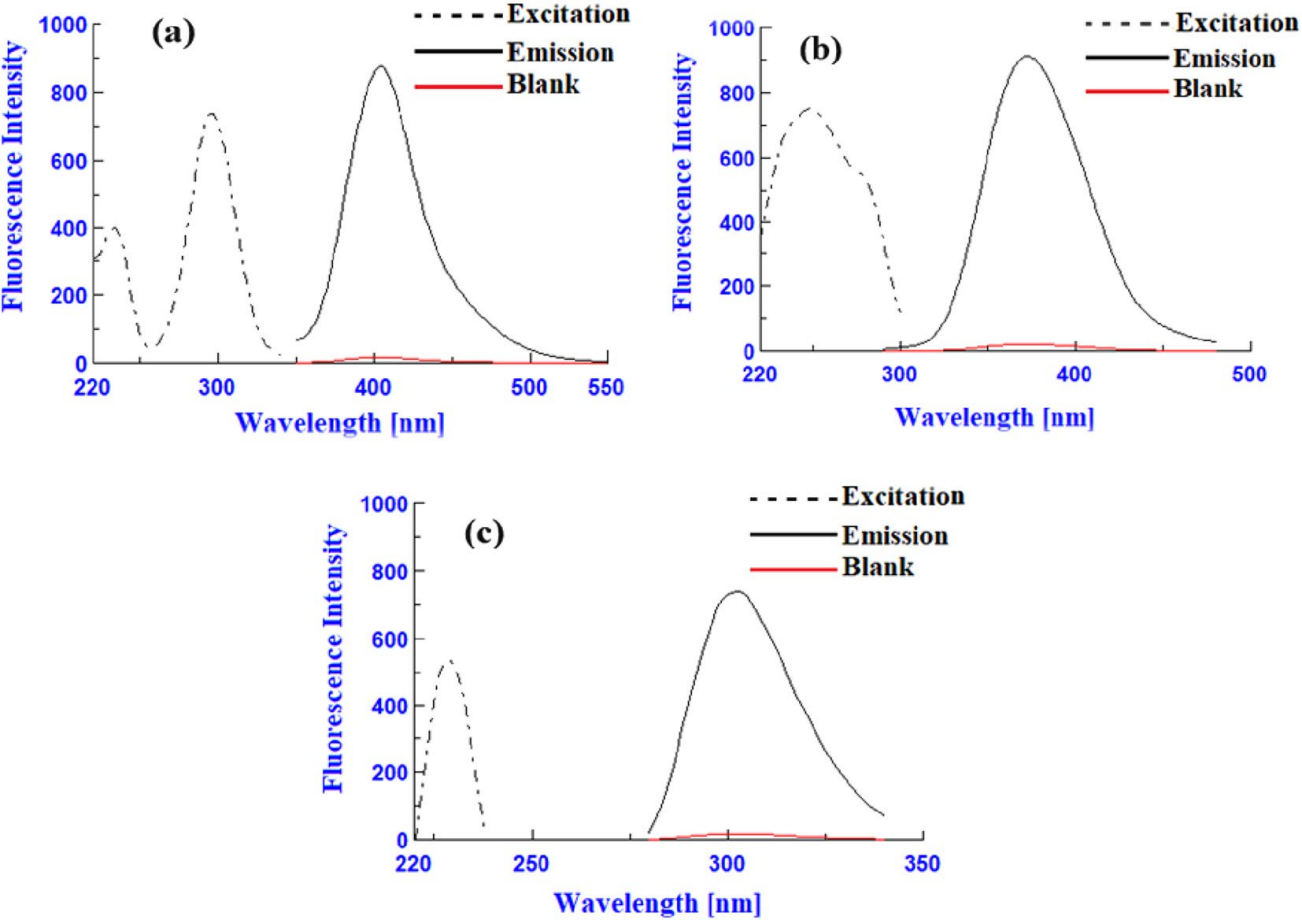
Excitation, emission, and blank spectra of ASA (**a**), OST (**b**), and MPL (**c**) in water.

**Figure 3:**
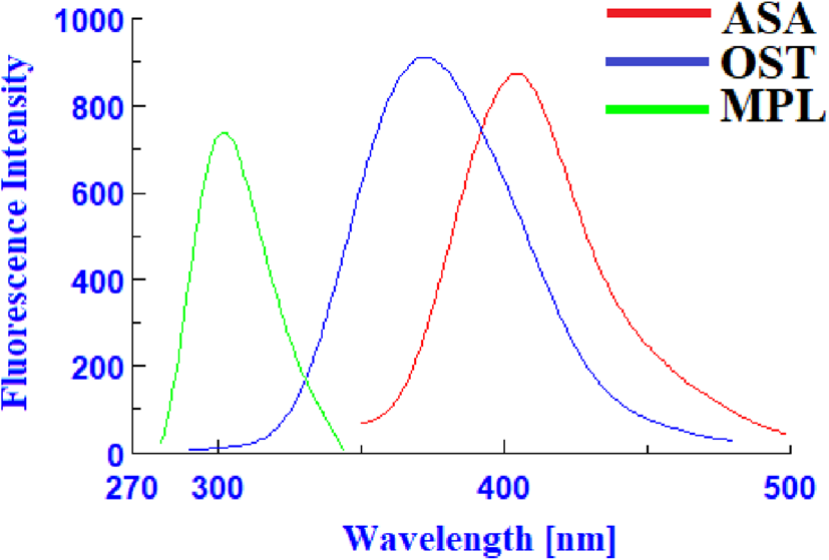
Emission spectra of ASA (1000 ng/mL), OST (800 ng/mL), and MPL (1100 ng/mL) in water.

**Figure 4:**
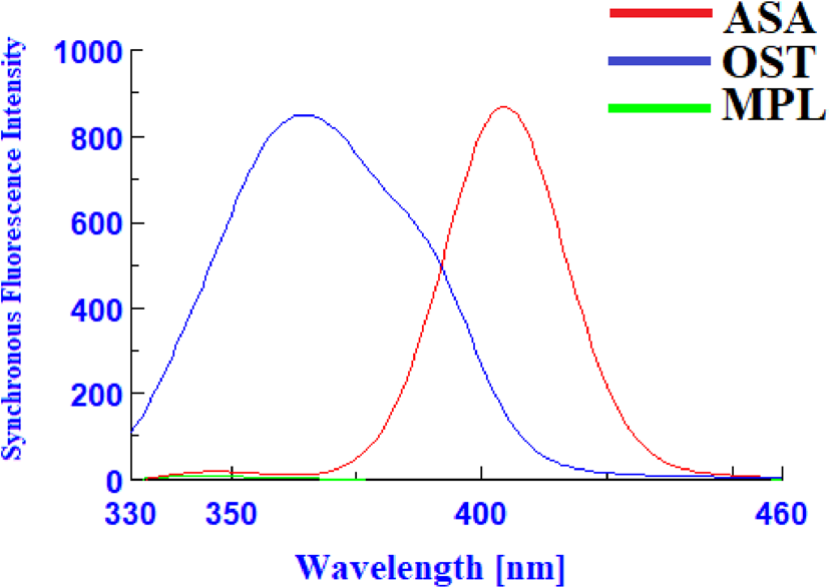
Synchronous fluorescence spectra of ASA (1000 ng/mL), OST (800 ng/mL), and MPL (1100 ng/mL) in water using Δλ = 110 nm.

**Figure 5:**
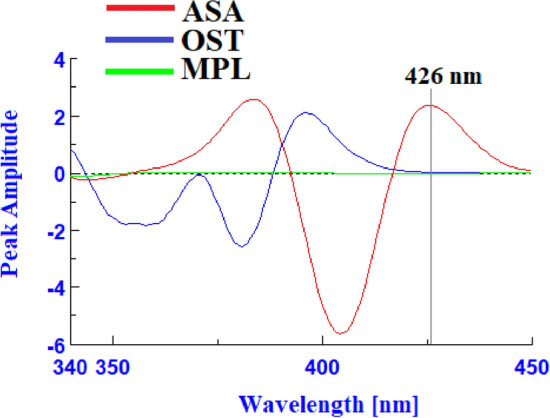
Second derivative synchronous fluorescence spectra of ASA (1000 ng/mL), OST (800 ng/mL), and MPL (1100 ng/mL) in water.

ASA, OST, and MPL had native emission signals at 404 nm, 372 nm, and 302 nm, when they were excited at 290 nm, 250 nm, and 230 nm, respectively (Fig. 2). The native fluorescence spectra indicated that there is no spectral overlap between the MPL emission spectra and those of ASA or OST, however the ASA and OST emission spectra overlapped (Fig. 3). As a result, MPL might potentially be detected directly from the native emission signals at 302 nm after excitation at 230 nm with no interference from ASA or OST. To improve the spectral resolution between ASA and OST spectra, the studied drugs were measured in synchronous fluorescence mode at various Δλ. Using Δλ = 110, ASA and OST had distinct synchronous fluorescence peaks at 404 nm and 364 nm, respectively, but MPL had no synchronous fluorescence responses (Fig. 4). OST synchronous fluorescence spectra were totally resolved from ASA synchronous fluorescence spectra at 364 nm, allowing OST to be determined directly from the synchronous fluorescence spectra at 364 nm with no interference from ASA or MPL. In contrast, ASA synchronous fluorescence spectra were not completely resolved for OST spectra, hindering direct determination of ASA using synchronous fluorescence spectra. The overlapping issue was not resolved by transforming the synchronous fluorescence spectra into a first-order derivative, whereas transforming the synchronous fluorescence spectra into a second-order derivative allowed for the determination of ASA at 426 nm with no interference from OST or MPL (Fig. 5).

### Method optimization

The method should be optimized for the variables that may affect the native and synchronous fluorescence spectra. Fixed concentrations of the studied drugs were utilized in the optimization trials, and one variable was altered while the others remained constant. The native fluorescence intensities of the studied drugs were compared using different diluting solvents. MPL and OST were found to have the greatest fluorescence intensities with water, but ASA had the highest fluorescence intensities with ethanol, followed by water. As a result, water was chosen as a diluting solvent. Additionally, the native fluorescence intensities of the studied drugs were examined using different buffer solution with varying pH and volumes. The highest signals of the tested medications were observed in 1.5 mL acetate buffer pH 5. For synchronous scanning, on the other hand, it is critical to choose the optimal ∆λ that provides the best spectral resolution and intensities. Therefore, we scanned the synchronous spectra of the medications over a variety of ∆λ values. The ∆λ = 110 was the optimum since it produced greater resolution with acceptable intensities.

### Validation

The suggested spectrofluorimetric approach was validated using ICH M10 on bioanalytical method validation guidelines.

#### Calibration curves and range

Calibration curves were created by graphing MPL fluorescence intensities at 302 nm, OST synchronous fluorescence intensities at 364 nm, and ASA second derivative peak amplitudes at 426 nm against the corresponding drug concentrations. For ASA, OST, and MPL, the calibration ranges (specified by the LLOQ and ULOQ) were 20–1000 ng/mL, 50–800 ng/mL, and 100–1400 ng/mL, respectively. The method showed accurate and precise linear relationship with coefficient of determination (r^2^) of 0.9997, 0.9994, and 0.9995 for ASA, OST, and MPL, respectively. The LLOQs was defined with the concentrations of the studied drugs that gives responses at least five times as the blank responses. The ULOQs was defined with the concentrations of the studied drugs that gives the highest fluorescence responses. The calibration curves parameters were listed in Table 1.

**Table 1:** Regression data for the determination of the studied drugs in plasma by the proposed method.

Parameters	ASA	OST	MPL
Wavelength (nm)	426	364	302
Linearity range (ng/mL)	20–1000	50–800	100–1400
LLOQ (ng/mL)	20	50	100
ULOQ (ng/mL)	1000	800	1400
Slope	0.0023	1.0471	0.6348
Intercept	0.1142	21.3428	49.9542
Coefficient of determination (r^2^)	0.9997	0.9994	0.9995

#### Selectivity

The capacity of the applied approach to selectively quantify the measured medications in the presence of matrix interferent was investigated by analysing blank sample prepared from pooled plasma collected from six separate sources. The interfering components were discovered to have no measurable fluorescence signal at the fluorescence responses of the investigated drugs (Fig. 2).

#### Accuracy and precision

The validity of the applied procedure for selective determination of the tested medications in spiked plasma sample was assessed by calculating the accuracy and precision of the QC samples made for each drug at four concentration levels within the calibration range. Each sample was measured in five replicates on the same day for intra-day accuracy and precision, and on 3 days for inter-day accuracy and precision. The accepted accuracy limit should be within ± 20% for the LLOQ and within ± 15% for the other QC levels. The accepted precision %CV shouldn’t exceed 20% for LLOQ and 15% for the other QC levels. Table 2 shows that all accuracy and precision calculations were within acceptable limits.

**Table 2:** Accuracy and precision of the assay in plasma.

Drug	Level	Added (ng/mL)	Analysis time	Found (mean ± SD, ng/mL)	Accuracy (mean%)		Precision (%CV)	
					Intra-day (n = 5)	Inter-day (n = 15)	Intra-day (n = 5)	Inter-day (n = 15)
ASA	LLOQ	20	Day 1	19.58 ± 0.57	97.91	98.33	2.89	2.43
			Day 2	19.91 ± 0.53	99.53		2.64	
			Day 3	19.51 ± 0.30	97.56		1.54	
	Low QC	50	Day 1	49.83 ± 1.21	99.66	98.62	2.44	2.30
			Day 2	48.96 ± 0.85	97.92		1.74	
			Day 3	49.14 ± 1.33	98.27		2.71	
	Medium QC	400	Day 1	398.57 ± 4.43	99.64	99.67	1.11	1.01
			Day 2	398.83 ± 5.13	99.71		1.29	
			Day 3	398.65 ± 3.26	99.66		0.82	
	High QC	800	Day 1	800.26 ± 9.09	100.03	99.78	1.14	0.96
			Day 2	796.35 ± 7.10	99.54		0.89	
			Day 3	798.00 ± 7.93	99.75		0.99	
OST	LLOQ	50	Day 1	48.64 ± 1.28	97.28	96.75	2.64	2.43
			Day 2	48.41 ± 1.16	96.82		2.39	
			Day 3	48.08 ± 1.28	96.15		2.67	
	Low QC	100	Day 1	98.44 ± 1.65	98.44	97.62	1.67	1.86
			Day 2	97.30 ± 1.48	97.30		1.52	
			Day 3	97.11 ± 2.30	97.11		2.37	
	Medium QC	350	Day 1	347.86 ± 4.71	99.39	99.72	1.35	1.16
			Day 2	349.96 ± 4.75	99.99		1.36	
			Day 3	349.19 ± 3.08	99.77		0.88	
	High QC	650	Day 1	649.45 ± 7.71	99.91	100.21	1.19	1.21
			Day 2	651.36 ± 9.05	100.21		1.39	
			Day 3	653.27 ± 8.08	100.50		1.24	
MPL	LLOQ	100	Day 1	96.49 ± 3.04	96.49	97.09	3.15	2.50
			Day 2	97.25 ± 1.84	97.25		1.89	
			Day 3	97.53 ± 2.71	97.53		2.78	
	Low QC	250	Day 1	249.64 ± 4.26	99.86	99.67	1.70	1.37
			Day 2	249.40 ± 2.88	99.76		1.15	
			Day 3	248.45 ± 3.68	99.38		1.48	
	Medium QC	600	Day 1	600.04 ± 6.83	100.01	99.83	1.14	1.08
			Day 2	599.13 ± 8.08	99.85		1.35	
			Day 3	597.76 ± 5.69	99.63		0.95	
	High QC	1200	Day 1	1198.06 ± 8.01	99.84	99.72	0.67	0.71
			Day 2	1193.30 ± 10.18	99.44		0.85	
			Day 3	1198.64 ± 8.17	99.89		0.68	

#### Matrix effect

The impact of unknown substances in the plasma matrix on drug fluorescence was assessed by examining low QC and high QC samples produced from six distinct plasma sources in triplicate. For each plasma matrix source, the accuracy and precision were determined. The accepted accuracy limit should be within ± 15%, and the precision (%CV) should not be larger than 15%. Table 3 reveals that the matrix has no effect on the fluorescence responses of the drugs.

**Table 3:** Matrix effect of the assay (n = 3).

Drug	Matrix	Low QC		High QC	
		Accuracy (mean%)	Precision (%CV)	Accuracy (mean%)	Precision (%CV)
ASA	Source 1	99.68	3.27	99.92	0.83
	Source 2	99.41	1.14	100.50	1.24
	Source 3	97.07	1.79	98.94	0.45
	Source 4	98.68	2.10	100.24	0.14
	Source 5	98.26	3.28	100.19	1.03
	Source 6	99.65	2.23	99.88	1.24
OST	Source 1	97.43	1.25	99.90	1.38
	Source 2	98.34	2.87	99.38	1.36
	Source 3	98.31	1.35	100.53	0.82
	Source 4	96.92	1.30	101.10	1.26
	Source 5	97.72	3.10	99.64	0.37
	Source 6	98.65	2.32	100.85	0.97
MPL	Source 1	97.71	3.22	100.09	0.39
	Source 2	100.46	1.44	99.79	0.91
	Source 3	99.99	1.54	99.34	0.81
	Source 4	97.14	2.05	99.29	0.91
	Source 5	100.08	0.95	100.10	0.43
	Source 6	99.39	1.60	99.70	1.04

#### Extraction recovery

To evaluate the efficiency of the extraction procedure, the extraction recovery was calculated by comparing the fluorescence responses of three QC concentration levels (low, medium, and high) prepared from six different plasma sources and spiked with the drugs standards before (pre-spiked) and after extraction (post-spiked). Table 4 reveals that the studied drugs had an acceptable extraction recovery.

**Table 4:** Extraction recovery of the assay (n = 3).

Drug	Matrix	Extraction recovery (%)		
		Low QC	Medium QC	High QC
ASA	Source 1	98.98	96.63	98.50
	Source 2	95.18	95.22	99.38
	Source 3	92.55	99.44	96.99
	Source 4	95.10	99.99	98.11
	Source 5	98.87	94.12	95.79
	Source 6	96.01	99.31	98.69
OST	Source 1	93.61	96.22	98.20
	Source 2	97.48	98.60	99.41
	Source 3	99.00	98.05	96.90
	Source 4	99.81	97.78	97.09
	Source 5	98.15	96.88	97.69
	Source 6	96.96	97.45	98.36
MPL	Source 1	94.65	96.85	99.11
	Source 2	96.32	99.02	98.20
	Source 3	96.48	95.97	99.37
	Source 4	97.00	97.82	96.84
	Source 5	93.83	97.63	96.99
	Source 6	94.99	99.64	97.27

#### Stability

Stability testing was performed to guarantee that the concentrations of the drugs under study did not change over the analysis duration and storage conditions. As a result, bench top stability for the analysis time (3 h) at RT was performed, as well as freeze–thaw matrix stability for three freezing–thawing cycles. For the stability experiments, fresh low and high QC samples generated in triplicate were employed. Table 5 shows that the drugs studied were stable on both the bench top and in freeze–thaw matrix stability tests.

**Table 5:** Stability of the developed method for the quantification of the studied drugs.

Drug	Bench top stability				Freeze–thaw matrix stability			
	Low QC		High QC		Low QC		High QC	
	Accuracy (mean%)	Precision (%CV)	Accuracy (mean%)	Precision (%CV)	Accuracy (mean%)	Precision (%CV)	Accuracy (mean%)	Precision (%CV)
ASA	97.35	2.01	99.66	0.94	96.89	1.88	96.88	1.09
OST	98.42	3.17	98.80	2.07	96.16	2.69	99.69	1.53
MPL	96.12	3.63	97.59	1.77	97.50	3.27	97.79	2.39

### Greenness examination

The greenness of the method was investigated using GAPI and AGREE tools. The GAPI tool utilize five pictograms and symbol. The pictograms represent the five stages of the analytical method: collection of the samples, preparation of the samples, reagent and solvent, apparatus, and type of the applied method. The symbol represents the type of analysis. Each pictogram represents a distinct feature, and each aspect was graded from green to yellow to red, indicating a low, medium, or high environmental effect. According to the GAPI assessment of the applied approach (Table 6), the method is very green, with 9 aspects assessed green and just one assessed red.

**Table 6:** Greenness evaluation of the proposed method.

GAPI	AGREE
	

The Analytical GREEnness (AGREE) tool, on the other hand, is a clock-like calculator pictogram with 12 assessment criteria that correspond to the 12 green analytical chemistry principles. Each assessment criterion was graded from 0 to 1, with a matching colour scale ranging from red to green. The overall score and colour were depicted in the center of the pictogram. According to the AGREE assessment of the applied approach (Table 6), the method achieved an overall score of 0.79 with a green colour, indicating that it is highly green.

## Conclusion

A simple, sensitive, and green spectrofluorimetric technique for detecting aspirin, olmesartan, and metoprolol in human plasma was developed in this study. The suggested methodology was verified using ICH M10 bioanalytical method validation criteria and proved effective for the measurement of the investigated drugs in plasma matrix with high accuracy and precision findings. Furthermore, the method received a high greenness score on both GAPI and AGREE greenness metrics.

## Data Availability

The datasets used and/or analyzed during the current study available from the corresponding author on reasonable request.
